# Effects of inadequate maternal dietary protein:carbohydrate ratios during pregnancy on offspring immunity in pigs

**DOI:** 10.1186/1746-6148-8-232

**Published:** 2012-11-28

**Authors:** Margret Tuchscherer, Winfried Otten, Ellen Kanitz, Maria Gräbner, Armin Tuchscherer, Olaf Bellmann, Charlotte Rehfeldt, Cornelia C Metges

**Affiliations:** 1 Research Unit Behavioural Physiology, Leibniz Institute for Farm Animal Biology (FBN), Dummerstorf, Germany; 2 Research Unit Genetics and Biometry, Leibniz Institute for Farm Animal Biology (FBN), Dummerstorf, Germany; 3 Leibniz Institute for Farm Animal Biology (FBN), Dummerstorf, Germany; 4 Research Unit Muscle Biology & Growth, Leibniz Institute for Farm Animal Biology (FBN), Dummerstorf, Germany; 5 Research Unit Nutritional Physiology “Oskar Kellner”, Leibniz Institute for Farm Animal Biology (FBN), Dummerstorf, Germany

## Abstract

**Background:**

Inadequate nutrition *in utero* may retard foetal growth and alter physiological development of offspring. This study investigated the effects of low and high protein diets fed to primiparous German Landrace sows throughout pregnancy on the immune function of their offspring at different ages. Sows were fed diets with adequate (AP, 12.1%; *n* = 13), low (LP, 6.5%; *n* = 15), or high (HP, 30%; *n* = 14) protein content, made isoenergetic by varying carbohydrate levels. Cortisol, total protein and immunoglobulin (IgG, IgM, IgA) concentrations were measured in the blood of sows over the course of pregnancy. Cortisol, total protein, immunoglobulins, lymphocyte proliferation, immune cell counts, and cytokines were assessed in the blood of offspring at baseline and under challenging conditions (weaning; lipopolysaccharide (LPS) administration).

**Results:**

In sows, the LP diet increased cortisol (*P* < 0.05) and decreased protein levels (*P* < 0.01) at the end of pregnancy. Immunoglobulin concentrations were decreased in LP (IgA) and HP piglets (IgG, IgM and IgA) on the first day of life (*P* < 0.05), whereas the number of lymphocytes and mitogen-induced lymphocyte proliferation of the piglets were unaffected by the maternal diet. Mortality during the suckling period was higher in LP piglets compared with AP and HP offspring (*P* < 0.01). Furthermore, LP piglets showed an elevated cortisol response to weaning, and in HP piglets, the CD4^+^ cell percentage and the CD4^+^/CD8^+^ ratio increased after weaning (*P* < 0.05). The lipopolysaccharide-induced rise of IL-6 was higher in LP (*P* = 0.09) and HP (*P* < 0.01) compared with AP piglets, and LP piglets displayed higher IL-10 levels than AP piglets (*P* < 0.05).

**Conclusions:**

Our results indicate that both low and high protein:carbohydrate ratios in the diet of pregnant sows can induce short-term as well as long-lasting effects on immune competence in piglets that may have serious consequences for host defence against bacterial pathogens.

## Background

Inadequate maternal nutrition and stress during gestation can affect the physiological development of offspring and may increase their susceptibility to diseases later in life [1,2]. Across species, including laboratory animals, non-human primates, and humans, reduced birth weight is a major outcome of disturbances during gestation [3] that may be associated with altered activity of the neuroendocrine system [4] and modified immune function in offspring [5]. Also in domestic pigs, prenatally stressed offspring displayed altered hypothalamic-pituitary-adrenal (HPA) axis regulation [6,7], suppressed humoral and cellular immune responses [8,9], increased cortisol levels after social mixing [10,11] and stronger fever and cytokine responses to an inflammatory stimulus [12,13]. Inadequate maternal dietary protein and/or carbohydrate levels during pregnancy in pigs have been shown to retard intrauterine growth resulting in low body weight at birth, and to affect body composition and properties of skeletal muscle and adipose tissue of the offspring [14-16]. The main metabolic effect in pregnant sows fed a high protein-low carbohydrate diet was a glucose and energy deficit, whereas a low protein-high carbohydrate diet resulted in a lack of indispensable amino acids, as recently reported by our group [17].

Yet, only few studies in rodents have examined the effects of imbalanced maternal protein nutrition throughout pregnancy on dam and offspring immunity. In rats, both low (4%) and high (20%) dietary protein levels during gestation led to alterations in plasma protein, albumin and γ-globulin levels of pregnant rats and their neonates compared with a 10% control diet [18]. Furthermore, moderate dietary protein restriction in pregnant rats impaired offspring thymocyte proliferation at birth and thymic and spleen lymphocyte proliferation at weaning [19]. Although there is increasing evidence that production characteristics in pigs may be affected by dietary protein imbalance during gestation [20], knowledge on the effects of inadequate maternal dietary protein levels on the developing immune system in porcine offspring is lacking. This is especially relevant because in addition to a low birth weight, altered immune reactivity in neonatal pigs is associated with a greater risk of postnatal mortality, a major concern with regard to animal welfare [21,22].

Moreover, exposure to adverse nutritional environments *in utero* has been shown to have programming effects on tissue function that may be related to elevated risk of metabolic, endocrine and cardiovascular disorders in adulthood [23,24]. For example, a low protein diet in rodent pregnancy has induced high blood pressure and renal dysfunction in the offspring [25], and prenatal high dietary protein exposure resulted in increased adiposity in young rats [26]. However, little is known regarding whether prenatal nutritional factors may also alter the physiological adaptive responses of offspring to stressful situations or immune challenges later in life.

In the present study, we used our recently developed model of intrauterine growth restriction in which dietary protein to carbohydrate ratios in pregnant primiparous sows are modulated [15]. The objective of this experiment was to investigate the effects of low (6.5%) and high (30%) protein:carbohydrate ratios in the diet of sows throughout pregnancy on the immune system of their offspring at different ages. To this end, the impact of the maternal diet on cortisol, protein and immunoglobulin levels in sow blood over the course of pregnancy was determined, and the immune systems of piglets were evaluated by measuring total serum protein and immunoglobulin levels (IgG, IgM, IgA), lymphocyte proliferation, proportions of circulating lymphocyte subpopulations and cytokine levels at baseline and under challenging conditions (weaning and LPS administration). The intensity of the challenge was also measured by determining the response of the HPA axis.

## Methods

All procedures including use and treatment of animals were in accordance with the German animal protection law and approved by the relevant authorities (Landesamt für Landwirtschaft, Lebensmittelsicherheit und Fischerei Mecklenburg-Vorpommern, Germany; LVL M-V/TSD/7221.3-1.1-006/04; LALLF M-V/TSD/7221.3-1.2-05/06; LALLF M-V/TSD/7221.3-1.2-013/06). The present experimental investigation is part of a comprehensive study recently described by Rehfeldt et al. [15].

### Animals and treatments

A total of 42 primiparous German Landrace sows and their litters, bred and raised in the experimental pig unit of our institute, were used for the experiment with 6 independent replicates. Housing and breeding management were as recently described in detail [15]. The sows were fed an isoenergetic corn-barley and soybean meal diet (~13.7 MJ ME/kg) containing an adequate (AP, 12.1%; n = 13), a low (LP, 6.5%; n = 15) or a high (HP, 30%; n = 14) protein level corresponding to protein:carbohydrate ratios of 1:5, 1:10.4, and 1:1.3, respectively, throughout gestation [15]. Diets were fed between 2.3 and 2.9 kg/d from early to late pregnancy to achieve an average target energy intake of ~34 MJ ME/d during gestation following the recommendations for primiparous sows [27]. The sows were fed twice daily, and water was provided *ad libitum*. Farrowing was induced at gestation day (GD) 114 as described previously [15].

After overnight food withdrawal, blood samples of sows were taken on GD −5, 24, 66 and 108 by jugular vein puncture. EDTA blood samples were centrifuged at 2000 × *g* for 15 min at 4°C to separate plasma, which was analysed for cortisol. Whole blood samples were allowed to clot for 4 h at room temperature and centrifuged at 1000 × *g* for 15 min at 4°C to obtain serum for analyses of total protein and immunoglobulins IgG, IgA and IgM. Plasma and serum samples were stored at −20°C until analysis.

**Figure 1:**
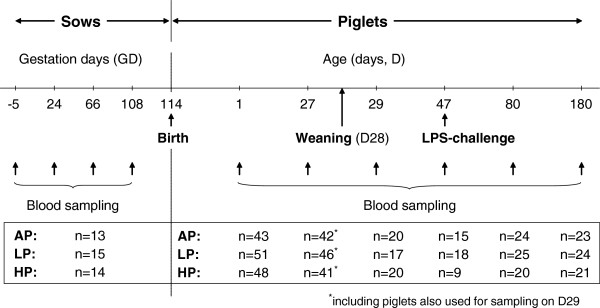
**Timeline: summary of blood sampling and distribution of animals in different diet groups. ** Sows were fed adequate (AP, 12.1%), low (LP, 6.5%) and high (HP, 30%) dietary protein levels throughout gestation.

Litter size, piglets born alive and dead, individual birth weights and sex of piglets were recorded at birth. Runt piglets weighing less than 800 g were excluded from further experiments. From each experimental litter, usually three to four piglets (the lightest one, 1–2 of medium weight, the heaviest one) were sampled between 24 to 36 h after birth (day 1 (D1): AP, n = 43; LP, n = 51; HP, n = 48). Three to four other piglets, in single cases only two piglets of each litter, were randomly assigned for sampling on D27 (AP, n = 42; LP, n = 46; HP, n = 41), and the remaining piglets were sampled on D80 (AP, n = 24; LP, n = 25; HP, n = 20) or D180 (AP, n = 23; LP, n = 24; HP, n = 21). Sex was almost equally distributed within diets. The timeline for blood sampling and distribution of animals in the different diet groups are summarised in Figure 1.

Piglets were cross-fostered within 48 h after birth to multiparous sows fed a standard diet during pregnancy (Provital RF R.324.0; Trede & Pein, Dammfleth, Germany). The litters were standardised to 11 piglets with piglets from foster sows. After birth, experimental sows and foster sows were fed a single standard lactation diet (Provital LAC R.325.0; Trede & Pein, Dammfleth, Germany). Male piglets were castrated at four days of age. The piglets were weaned at D28 and housed in groups of four littermates per pen (2.5 m x 1.8 m) in a post-weaning room until D32. Thereafter, they were transferred to single-housing cages for the rest of the experimental period. Piglets were offered a commercial pellet diet from an automatic feeder. Food and water were provided *ad libitum*.

Blood samples from the offspring were taken while animals were in a supine position by anterior vena cava puncture on D1, D27, D29 and D47 as well as by jugular vein puncture on D80 and D180 (the whole procedure lasted approximately 1 min). Blood samples without anti-coagulant were treated as described above to obtain serum for analyses of total protein and immunoglobulins IgG, IgM and IgA (D1, D27, D80 and D180). Additionally, heparinised blood samples were stored on ice until processing for the proliferation assay (D1 and D27).

One day before (D27) and one day after weaning (D29), EDTA-plasma samples were used for cortisol analyses, and heparinised blood samples were collected and stored on ice until processing for the proliferation assay and flow cytometric analysis (AP, n = 20; LP, n = 17; HP, n = 20). On D47, a subset of piglets (AP, n = 15; LP, n = 18; HP, n = 9) received an intraperitoneal (i.p.) injection of LPS from *Escherichia coli* (serotype O111:B4, Sigma Chemical Company, St. Louis, MO, USA) at a dose of 100 μg/kg of body weight dissolved in 2 mL sterile saline. Blood samples were taken immediately before LPS injection (0h, baseline for all measures), 1 h later for analysis of TNF-α, and 3 and 6 h later to measure cortisol and cytokines (TNF-α, IL-6 and IL-10). Blood samples were treated as described previously.

### Laboratory analyses

Plasma cortisol concentrations were analysed in duplicates using a commercially available ^125^I-RIA kit (DSL, Inc., Sinsheim, Germany) according to the manufacturer’s instructions. The test sensitivity was 8.1 nmol/L, and intra- and inter-assay coefficients of variation were 8.2% and 9.8%, respectively. Total protein content in serum was determined by the biuret-method (Bioquant® Protein 110307; Merck, Darmstadt, Germany). Concentrations of immunoglobulins IgG, IgA and IgM were determined in duplicate with porcine specific enzyme-linked immunosorbent assays (ELISA) according to the manufacturer’s instructions (Bethyl, Laboratories Inc., Montgomery, TX USA). The intra-assay and inter-assay coefficients of variation for the ELISAs were <5% and <10%, respectively. TNF-α and IL-10 concentrations were analysed in plasma samples using commercially available pig ELISA kits (Biosource Invitrogen, Carlsbad, California, USA) according to the manufacturer’s instructions. The sensitivity of the TNF-α assay was 3 pg/mL, and intra- and inter-assay coefficients of variation were 6.2% and 8.2%, respectively. The detection limit of the IL-10 assay was 3 pg/mL. Intra- and inter-assay coefficients of variation were 6.3% and 9.4%, respectively. Plasma concentrations of IL-6 were determined with pig IL-6 ELISA kits (R&D Systems Inc., Minneapolis, MN, USA) according to the manufacturer’s instructions. The sensitivity of this assay was 10 pg/mL, and the intra- and inter-assay coefficients of variation were 3.5% and 8.1%, respectively.

The mitogens Concanavalin A (5 μg/mL; ConA) and lipopolysaccharide (10 μg/mL; LPS) were used in lymphocyte proliferation/viability assays as previously described [28]. Briefly, peripheral blood mononuclear cells (PBMC) were isolated from heparinised blood by density gradient centrifugation, and the cell concentration was adjusted to 5 × 10^6^ cells/mL in complete RPMI-1640 medium (Sigma-Aldrich, Deisenhofen, Germany). PBMCs were incubated in 96-well microplates for 72 h in a 5% CO_2_ humidified incubator at 37°C. Cell proliferation/viability was evaluated using the 3-[4,5-dimethyldiazol-2-yl]-2,5 diphenyl tetrazolium bromide (MTT) assay (Roche Diagnostics, Mannheim, Germany). The optical density (O.D.) was measured by a microplate reader (Dynatech, Denkendorf, Germany) using a test wavelength of 550 nm and a reference wavelength of 690 nm. The results were expressed as a proliferation index (PI), which was calculated as follows: PI = O.D. in the presence of mitogen/O.D. in the absence of mitogen.

Immunofluorescence double staining for flow cytometry was performed by incubating 200 μL of the PBMC suspension (1 × 10^6^ cells) with 10 μL of fluorescein isothiocyanate (FITC)-conjugated mouse-anti-porcine CD4α (IgG2b isotype) and 10 μl R-phycoerythrin (RPE)-conjugated mouse-anti-porcine CD8α (IgG2a isotype) monoclonal antibodies (AbD Serotec, Oxford, UK) in the dark at room temperature for 30 min. Excess antibodies were removed by washing plates twice with PBS/1% fetal bovine serum (FBS) and centrifuging at 180 × g for 5 min at 20°C. The cell pellet was resuspended in 1 mL of PBS/1% FBS for immediate analysis using an EPICS XL flow cytometer (Beckman Coulter, Krefeld Germany). The cells were gated on previously established bitmaps based on the forward and side scatter characteristics of each population. The proportion of cells was calculated after acquisition of at least 6000 events as described by Löhrke et al. [29]. To estimate the specific binding of mouse monoclonal antibodies to cell surface of lymphocytes mouse-IgG2b negative control-FITC and mouse-IgG2a negative control-RPE (Serotec, Oxford, UK) were used.

### Statistical analysis

Statistical analyses were performed using the SAS System for Windows, release 9.2 [30]. Data were evaluated by analysis of variance (ANOVA) using the Mixed procedure. The model for blood parameters of sows comprised the fixed effects diet (AP, LP, HP), replicate (1 to 6), repeated factor time (GD − 5, GD24, GD66, GD108) and all two-way interactions. Gestation length and litter data were analysed with the model comprising the fixed effects diet, replicate and the interaction diet × replicate. The mixed model for birth weight and early weight gain of piglets included the fixed effects diet, sex (male, female) and replicate, the corresponding 2-way interactions and the random effect of sow. Frequencies of stillborn and dead piglets per litter were analysed by fitting a logistic model using the GLIMMIX procedure. The model included the fixed effects diet, replicate and the interaction diet × replicate. The model for basal blood parameters in offspring comprised the fixed effects diet, age (D1, D27, D80, D180), sex, replicate, all two-way interactions between the fixed effects and the random sow effect. The model for weaning data and for measurement with LPS challenge in piglets included the fixed effects diet, sex, replicate, sampling time as repeated factor (for weaning: D27, D29 and for LPS challenge: 0 h, 1 h, 3 h, 6 h), all two-way interactions between these fixed effects and the random sow effect. In addition, least-squares means (LS-means) and their standard errors (SE) were computed for each fixed effect in the models, and all pairwise differences of LS-means were tested by the Tukey-Kramer procedure. Correlations between plasma cortisol levels in sows on GD108 and immune parameters in their neonatal offspring were estimated by Spearman’s rank correlation coefficients within each diet group using the Corr procedure. Effects and differences were considered significant if *P* < 0.05.

## Results

### General observations

No effects of diet were found on the gestation length, the total number born or the frequency of stillborn piglets (*P* > 0.3; data not shown). Further, the litter size was not influenced by diet (AP: 11.5 ± 0.7; LP: 12.7 ± 0.7; HP: 11.5 ± 0.8; *P* = 0.42). However, the maternal diet significantly affected the weight of piglets at birth. Both the LP and the HP diet caused a reduction in offspring birth weight compared to control AP piglets (AP: 1.35 ± 0.04 kg; LP: 1.20 ± 0.03 kg; HP: 1.23 ± 0.03 kg; *P* < 0.05), which is consistent with the results obtained for all piglets born^(15)^. LP piglets gained less weight compared with AP and HP piglets during the first day of life (AP: 78 ± 15 g; LP: 29 ± 14 g; HP: 85 ± 15 g; *P* = 0.05). Piglet mortality during the suckling period was higher in the LP than in the AP and HP groups (AP: 3.0 ± 1.7%; LP: 11.2 ± 1.7%; HP: 4.0 ± 1.8%; *P* < 0.01).

### Effects of diet on sows

Plasma cortisol concentrations of primiparous sows were affected by diet and time (Table 1). HP sows had lower cortisol levels than AP (*P* < 0.01) and LP sows (*P* < 0.05). The Tukey-Kramer means separation procedure indicated that the cortisol concentration in the plasma from LP sows was significantly higher compared with HP sows on GD108 (*P* < 0.05). In AP and LP sows, cortisol levels increased from early to late pregnancy (*P* < 0.05). In this subset of sows, serum protein concentrations tended to be affected by diet, but were significantly influenced by time and diet × time interaction (Table 1). On GD108, protein concentrations were significantly lower in LP than in AP (*P* < 0.01) and HP sows (*P* < 0.001). In addition, the protein levels decreased within the LP group at the end of pregnancy (*P* < 0.01), whereas the protein levels increased within the HP group from GD24 to GD66 (*P* < 0.05). Serum immunoglobulin levels were affected by time during pregnancy (*P* < 0.001), but not by pregnancy diet (Table 1). Independent of the diet, IgG and IgM levels increased from GD24 to GD66 (*P* < 0.01). In AP and LP sows, serum IgA decreased from GD − 5 to GD24 (*P* < 0.01). Furthermore, ANOVA revealed a significant diet × time interaction for IgA levels.

**Table 1:** Cortisol, total protein and immunoglobulin concentrations of sows on gestation days −5, 24, 66 and 108

		**Time**					***P*-values**		
	**Diet**	**GD − 5**	**GD24**	**GD66**	**GD108**	**SE**	**Diet**	**Time**	**Diet × Time**
Cortisol	Adequate	64.8^a^	63.3^a^	86.8	95.8^b^	6.9			
(nmol/L)	Low	51.4^a^	65.4^a^	72.4^a^	101.9^A,b^	6.4	0.009	< 0.001	0.249
	High	52.5	50.0	62.3	73.9^B^	6.8			
Total protein	Adequate	77.7	75.1	79.2	76.6^A^	1.3			
(mg/mL)	Low	79.3^a^	75.1^a^	77.0^a^	68.8^B,b^	1.3	0.082	< 0.001	< 0.001
	High	79.5	75.1^a^	80.4^b^	78.5^A^	1.4			
IgG (mg/mL)	Adequate	15.9	12.9^a^	17.1^b^	14.4	0.8			
	Low	14.4	13.5^a^	17.7^b^	16.4	0.7	0.697	< 0.001	0.241
	High	14.9	13.2^a^	17.6^b^	16.8	0.8			
IgM (mg/mL)	Adequate	6.10	5.26^a^	7.70^b^	7.22	0.62			
	Low	6.42	6.10^a^	8.01^b^	7.54	0.58	0.519	< 0.001	0.481
	High	6.44	6.89^a^	8.48^b^	7.98	0.64			
IgA (mg/mL)	Adequate	1.15^a^	0.63^b^	0.83	1.03	0.12			
	Low	1.21^a^	0.85^b^	1.11	1.10	0.12	0.517	< 0.001	0.012
	High	0.90	0.81	0.93	1.06	0.13			

*GD*, gestation day.

Sows were fed diets with adequate (12.1%, *n* = 13), low (6.5%, *n* = 15) and high (30%, *n* = 14) protein levels throughout gestation.

Data are expressed as least-squares means (LS-means) ± standard errors (SE).

*P* values for effects of diet, time and diet × time interaction.

^A,B^Within parameter and time, LS-means with unlike capital superscript letters were significantly different between diets (*P* < 0.05; Tukey-Kramer test).

^a,b^Within a row, LS-means with unlike lower case superscript letters were significantly different between times (*P* < 0.05; Tukey-Kramer test).

### Effects of maternal diet on offspring

**Figure 2:**
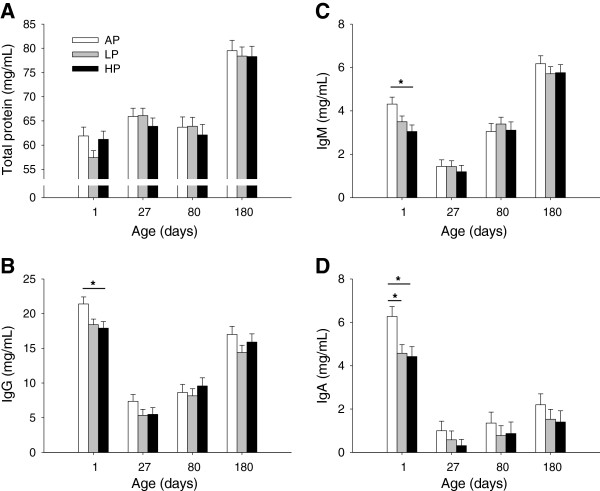
**Total serum protein (A) and immunoglobulin IgG (B), IgM (C) and IgA (D) concentrations in pigs.** Piglets were born to sows fed adequate (AP, 12.1%), low (LP, 6.5%) and high (HP, 30%) dietary protein levels throughout gestation. Blood samples were taken on D1 (AP, n = 43; LP, n = 51; HP, n = 48), D27 (AP, n = 42; LP, n = 46; HP, n = 41), D80 (AP, n = 24; LP, n = 25; HP, n = 20) and D180 (AP, n = 23; LP, n = 24; HP, n = 21). Data are expressed as LS-means + SE. Significant differences between diet groups are indicated by asterisk (* *P* < 0.05; Tukey-Kramer test).

Maternal diet did not affect the concentrations of serum total protein (*P* = 0.75) or immunoglobulins in the offspring (*P* > 0.2) over all ages examined. There were lower protein levels on D1, D27 and D80 compared with D180 for all pigs (*P* < 0.001, Figure 2A). We also found significant differences in the concentrations of immunoglobulins between the diet groups on the first day of life. At D1, the IgG and IgM levels in HP piglets were lower than in AP piglets (*P* < 0.05, Figure 2B and C, respectively), and the IgA levels were lower in both LP and HP piglets compared with AP piglets (*P* < 0.05, Figure 2D). Concentrations of IgG, IgM and IgA were significantly affected by age (*P* < 0.001), with similar patterns in all dietary groups. The lowest immunoglobulin levels were measured in piglets on D27 compared with D1, D80 and D180 (*P* < 0.001).

Further, there was no significant effect of maternal diet on the number of PBMCs or the mitogen-induced PBMC proliferation in response to either ConA or LPS in piglets at D1 and D27 of age (*P* > 0.2; data not shown). The factor sex and the interaction diet × age had no significant effects on any of the traits of the offspring that were investigated (*P* > 0.12).

### Relationship between cortisol levels in sows and immune parameters in their neonatal offspring

To investigate the relationships between plasma cortisol levels in sows on GD108 and immune parameters in their neonatal offspring, Spearman’s rank correlation coefficients were estimated within each diet group (AP: n = 41; LP: n = 49; HP: n = 46). In the LP and HP groups, negative correlations were found between the cortisol levels in sows at the end of pregnancy and total protein concentrations in the serum of piglets on the first day of life (rs = −0.306, *P* < 0.05, and rs = −0.378, *P* < 0.01, respectively), whereas no significant correlation was observed in the AP group (rs = −0.102, *P* = 0.52). There were also negative correlations between the cortisol levels of sows and IgA levels of piglets in both the LP and HP groups (rs = −0.374, *P* < 0.01, and rs = −0.443, *P* < 0.01, respectively), but not in the AP group (rs = +0.107, *P* = 0.52). In addition, the cortisol levels of sows and the IgM concentrations in piglets were negatively correlated in the LP group (rs = −0.481, *P* < 0.001), but were not in the AP and HP groups (rs = −0.201, *P* = 0.21, and rs = −0.198, *P* = 0.19, respectively). No further significant relationships were found between the cortisol levels in late-pregnant sows of the three diet groups and immune parameters in neonatal piglets (*P* > 0.2).

### Effects of maternal diet on the response to weaning stress

**Figure 3:**
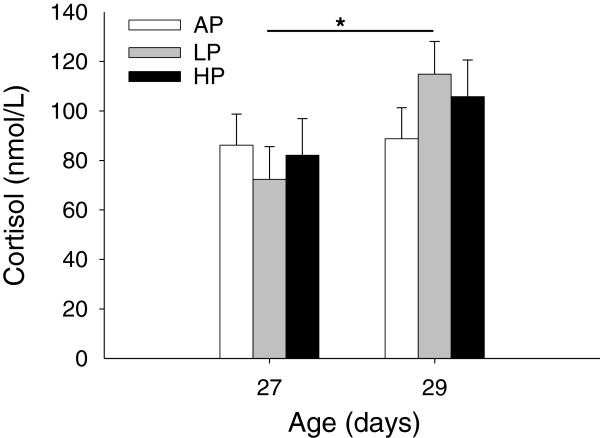
**Plasma cortisol concentration in weaned piglets. **Piglets were born to sows fed adequate (AP, 12.1%), low (LP, 6.5%) and high (HP, 30%) protein levels throughout gestation. Blood samples were taken before (D27) and after weaning (D29). Data are expressed as LS-means + SE (AP, n = 20; LP, n = 17; HP, n = 20). Significant differences between diet groups are indicated by asterisk (* *P* < 0.05; Tukey-Kramer test).

The plasma cortisol level was not affected by maternal diet and the interaction diet × sampling time (*P* > 0.14), but sampling time had a significant effect (*P* < 0.01). The Tukey-Kramer means separation test showed that only the LP piglets displayed a significantly higher cortisol level one day after weaning than before (*P* < 0.05, Figure 3).

**Figure 4:**
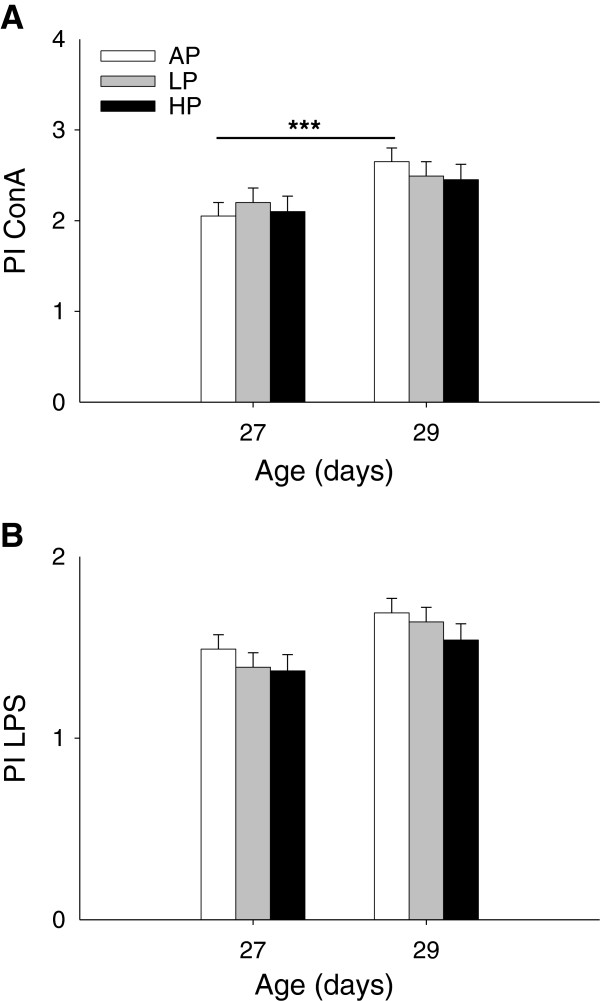
**Lymphocyte proliferation indices (PI) in response to ConA (A) and LPS (B) in weaned piglets.** Piglets were born to sows fed adequate (AP, 12.1%), low (LP, 6.5%) and high (HP, 30%) protein levels throughout gestation. Blood samples were taken before (D27) and after weaning (D29). Data are expressed as LS-means + SE (AP, n = 20; LP, n = 17; HP, n = 20). Significant differences between diet groups are indicated by asterisks (*** *P* < 0.001; Tukey-Kramer test).

The lymphocyte proliferation in response to both mitogens ConA and LPS was not influenced by maternal diet and diet × sampling time interaction (*P* > 0.21). There was an effect of sampling time for proliferation indices in mitogen-stimulated cultures (*P* < 0.01). The Tukey-Kramer procedure revealed in AP piglets that the ConA-stimulated lymphocyte proliferation was significantly higher on D29 than D27 (*P* < 0.001, Figure 4A).

**Figure 5:**
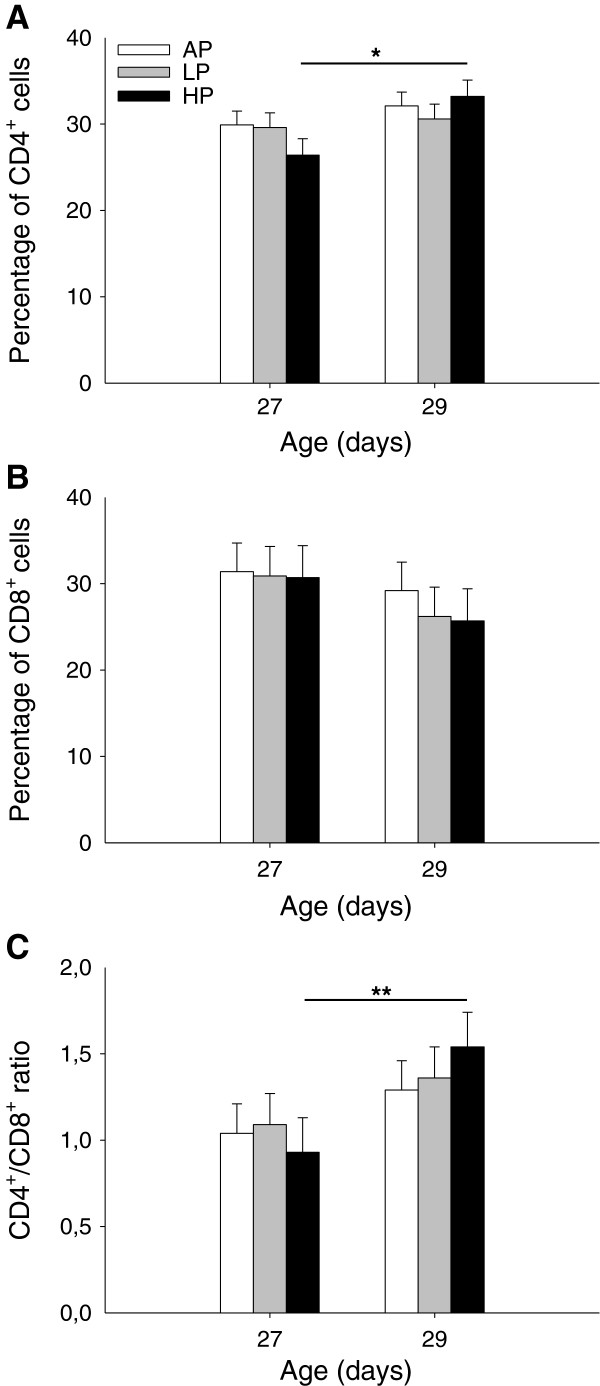
**Percentages of peripheral blood CD4+ cells (A), CD8+ cells (B) and CD4+/CD8+ ratios (C) in weaned piglets. **Piglets were born to sows fed adequate (AP, 12.1%), low (LP, 6.5%) and high (HP, 30%) protein levels throughout gestation. Blood samples were taken before (D27) and after weaning (D29). Data are expressed as LS-means + SE (AP, n = 20; LP, n = 17; HP, n = 20). Significant differences between diet groups are indicated by asterisks (* *P* < 0.05, ** *P* < 0.01; Tukey-Kramer test).

No significant effects of maternal diet and interaction diet × sampling time were found for the percentages of CD4^+^ and CD8^+^ cells or their ratio (*P* > 0.12), but these blood parameters were affected by sampling time (*P* < 0.01). For the percentage of CD4^+^ cells and the CD4^+^/CD8^+^ ratio, the Tukey-Kramer test indicated an increase in HP piglets (*P* < 0.05, Figure 5A and *P* < 0.01, Figure 5C, respectively). None of these variables was significantly affected by sex (*P* > 0.28).

### Effects of maternal diet on offspring response to LPS challenge

**Figure 6:**
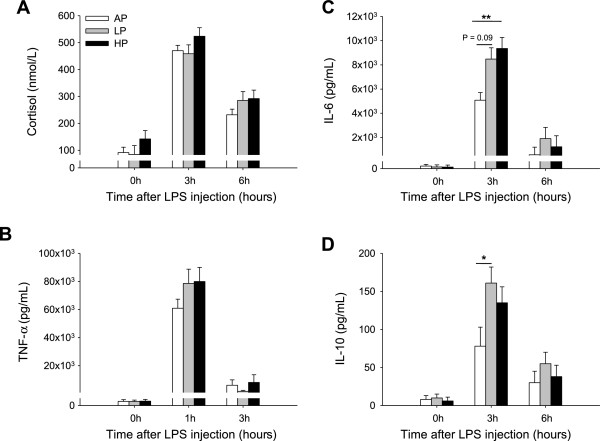
**Plasma cortisol (A), TNF-α (B), IL-6 (C) and IL-10 (D) concentrations in pigs challenged with LPS. **Piglets were born to sows fed adequate (AP, 12.1%), low (LP, 6.5%) and high (HP, 30%) protein levels throughout gestation. On D47, piglets received an i.p. injection of LPS from *Escherichia coli *(serotype O111:B4) at a dose of 100 μg/kg of body weight. Blood samples were taken before (0 h) and after LPS challenge (1 h, 3 h, 6 h). Data are expressed as LS-means + SE (AP, n = 15; LP, n = 18; HP, n = 9). Significant differences between diet groups are indicated by asterisks (* *P* < 0.05, ** *P* < 0.01; Tukey-Kramer test).

Intraperitoneal injection of 100 μg LPS/kg body weight induced significant increases in the plasma concentration of TNF-α 1 h later (Figure 6B) and in concentrations of cortisol (Figure 6A), IL-6 (Figure 6C) and IL-10 (Figure 6D) 3 h after LPS in piglets on day 47 (*P* < 0.001). However, only IL-6 concentration was influenced by both maternal diet and diet × sampling time interaction (P < 0.05). Three hours after the challenge, LP and HP piglets responded to LPS with higher IL-6 concentrations than AP piglets (P = 0.09 and P < 0.01, respectively). Furthermore, LP piglets showed higher IL-10 levels compared with AP piglets (P < 0.05). No significant effects of sex were observed (*P* > 0.21).

## Discussion

In animals and humans, a number of studies have indicated that dietary composition plays an important role in the immune system [31,32]. Increased attention is now being focused on the effects of nutritional factors during pregnancy on immune competence and health of the offspring. The present study demonstrates for the first time that both low and high protein:carbohydrate ratios in the diets fed to primiparous sows throughout pregnancy affect baseline immune parameters in neonatal offspring and modify immune responses to challenges later in life.

In late pregnancy, the LP diet caused lower protein concentrations in serum compared with AP and HP sows, an effect which is manifested by protein malnutrition [33,34]. As also shown in our investigation, concentrations of immunoglobulins are often unaffected by this type of malnutrition [32]. Moreover, the changes in IgG, IgM and IgA serum levels we observed in sows at different stages of pregnancy had similar patterns to those previously described by Klobasa et al. [35].

Because of the structure of the epitheliochorial porcine placenta, neonatal piglets acquire maternal immunity soon after birth by absorbing intact immunoglobulins from ingested colostrum for humoral immune protection. The amount of immunoglobulin uptake by the suckling piglet mainly depends on the concentration of immunoglobulins in colostrum, the intestinal capacity to absorb macromolecules prior to gut closure, the time to first suckle, and the colostrum intake [36,37]. In the present study, piglets born to sows receiving limited or excess dietary protein during pregnancy displayed lower serum immunoglobulin levels on the first day of life compared with those from mothers fed an adequate protein diet. Indeed, we did not find significant differences in colostral immunoglobulin concentrations between the sows fed different dietary protein levels [15]. The majority of prenatal stress research indicates that physiological alterations in prenatally stressed offspring can be attributed to the action of maternal glucocorticoids [2,38]. Previous studies in pigs have shown that prenatal stress during the late phase of gestation also decreased the circulating IgG levels in neonates [8,39]. An increase in maternal cortisol during late gestation may accelerate foetal gut maturation and thereby impair the acquisition of colostral immunoglobulins after birth [40]. Interestingly, although cortisol levels were significantly increased only in LP sows at the end of pregnancy, we found strong inverse relationships between the plasma cortisol levels in both LP and HP sows on D108 of gestation and the serum levels of total protein and IgA in their offspring on the first day of life. Thus, an altered permeability of the gut in neonates of sows fed diets with inadequate protein levels could explain the decreased immunoglobulin concentrations in the piglets*.* In a previous experiment using the same dietary treatments as in this study, we found that both LP and HP diets alter materno-foetal HPA regulation [41]. Thus, it appears that maternal glucocorticoids are involved in mediating the effects of imbalanced nutrition during pregnancy in pigs on the immunity of offspring. Furthermore, a suboptimal intake of colostrum may result in an inadequate transfer of maternal immunoglobulins to the newborn and thereby also contribute to pre-weaning mortality [42]. Although piglets of the LP and HP groups showed lower serum immunoglobulin levels in the early postnatal period, we detected a higher mortality rate only in the LP group during the suckling period. As discussed by Le Dividich et al. [36], reduced immunoglobulin concentrations do not always lead to an increased risk of mortality if piglets receive an adequate intake of colostrum. While early postnatal vitality was not measured in our study, LP piglets exhibited the lowest muscle mass at birth [16], and we found that their early weight gain was significantly lower than that of AP and HP piglets. From these results, we hypothesise that LP piglets may have consumed less colostrum over the first hours after birth, which, together with their low birth weight, decreased vitality and increased pre-weaning mortality risk. However, besides immunoglobulins, it is known that several other components of colostrum and milk such as hormones, growth factors and cytokines may play an important role in the physiological development and immune protection of neonatal piglets [36,43,44]. A major finding from the present study is that both limited and excess protein in the maternal diet during pregnancy caused reduced humoral immunity in neonatal piglets that was not evident in older pigs. Indeed, these transient early modifications could also alter the immune-reactivity towards infections later in life [45].

Previous studies in pigs have reported that gestational stress in sows may activate the HPA axis in offspring during novel or challenging situations [10,11] and may also affect the immunity of neonatal pigs in response to stressful stimuli [8,46]. In our study, piglets from sows fed diets with limited protein levels during pregnancy showed increased basal cortisol levels one day after weaning, whereas cortisol levels in HP piglets were only slightly increased or unaltered in AP piglets. After LPS challenge, there was no effect of inadequate dietary protein to carbohydrate ratios on cortisol release. This finding is in accordance with the results of de Groot et al. [12] and Couret et al. [9], where prenatal stress also did not affect cortisol levels of piglets in response to LPS. The differences in cortisol release after weaning and LPS administration in the present study may be due to the different nature of the stressors. It seems that the HPA axis of LP piglets is more sensitive to weaning stress with abrupt social, nutritional, and environmental changes, whereas cortisol release after LPS challenge reflects an immunological stress. Therefore, we conclude that elevated cortisol concentrations in LP sows at the end of pregnancy can affect HPA activity in offspring, especially in response to a multifactorial stress such as weaning.

Our results revealed a significant increase in ConA-stimulated cell proliferation after weaning in piglets of AP sows that may indicate a more effective cellular immune response [47,48]. Together with the unaltered cortisol level, the higher *in vitro* lymphocyte function in AP piglets may be considered as a greater ability to cope with weaning stress [49]. Furthermore, the percentage of CD4^+^ cells and thereby the CD4^+^/CD8^+^ ratios were significantly increased after the stress of weaning in HP piglets only. These findings are comparable with frequently reported changes in lymphocyte subpopulations after sustained stress. A long-term dexamethasone treatment of weanling piglets resulted in a higher percentage of CD4^+^ cells [50], and chronic stress in sheep and humans has been shown to increase the CD4^+^/CD8^+^ ratio in peripheral blood [51-53]. The rise in CD4^+^/CD8^+^ ratio may reflect recovery of immune competence in an attempt to restore homeostasis following a stressful event. Although some caution must be exercised in interpreting our results, the changes in distribution of CD4^+^ and CD8^+^ cells in HP piglets could indicate the extent of T-lymphocyte recovery due to the severity of weaning stress perceived by these piglets.

In the present study, pigs of all diet groups responded to intraperitoneal LPS administration with elevations in cortisol and cytokine levels in similar temporal patterns that were appropriate for acute gram-negative infection [54,55]. However, the levels of circulating IL-6 were higher in LP and HP piglets compared to AP piglets, and LP piglets additionally showed a higher increase in IL-10. From previous research in pigs it is known that prenatal stress may also alter physiological responsiveness of offspring to an immune stimulation with LPS. De Groot et al. [12] suggested that the non-specific inflammatory response to an LPS challenge increased in piglets from sows treated with cortisol during pregnancy, and it was recently shown that maternal restraint stress during gestation in pigs enhanced the magnitude of the TNF-α and IL-6 responses to LPS in the offspring [13]. In our study, the increased inflammatory cytokine responses in piglets exposed to inadequate maternal protein-to-carbohydrate levels *in utero* confirm these results and support the hypothesis that maternal stress or malnutrition during pregnancy can influence aspects of immune responses in the offspring to challenges.

In humans, elevated levels of IL-6, a predominantly pro-inflammatory cytokine, have been identified as a marker of disease risk and dysregulated inflammation [56,57]. Focussing on the highly increased IL-6 levels in LP and HP offspring 3 h after LPS injection, these piglets appear to be more susceptible to bacterial endotoxin exposure with an increased risk of an acute systemic inflammatory response syndrome [58]. This interpretation may be supported by the magnitude of TNF-α response observed in these pigs 1 h after LPS. Moreover, the higher release of peripheral IL-10 in LP and HP pigs at the same time as IL-6 may emphasise the crucial role of IL-10 in regulating inflammatory responses [59,60]. Although the LPS challenge is a simplified inflammation model, the present results suggest that such alterations in cytokine release can be disadvantageous for recovery from frequently occurring bacterial infections of the gastrointestinal and respiratory system in pigs.

## Conclusions

The present results demonstrate that both low and high protein:carbohydrate ratios in the diets fed to pregnant sows moderately decrease humoral immunity in neonatal offspring, modify adaptive responses to cope with weaning stress in piglets and cause dysregulation in inflammatory cytokine responses to acute LPS challenges later in life. Thus, inadequate protein and carbohydrate supplies during pregnancy can induce short-term as well as long-lasting effects on immune responses in piglets, and thereby may impair defences against potential pathogens in the offspring. The present findings highlight the importance of adequate maternal macronutrient ratios during foetal life to promote the immune competence and adaptive stability of progeny and to improve animal health and welfare.

## Abbreviations

AP: Adequate protein; ConA: Concanavalin A; GD: Gestation day; HP: High protein; LP: Low protein; LPS: Lipopolysaccharide; PBMC: Peripheral blood mononuclear cells.

## Competing interests

The authors declare that they have no competing interests.

## Authors’ contributions

C.C.M., C.R., W.O. and M.T. designed the study, M.T., W.O., E.K., M.G. and O.B. conducted the experiment, A.T. performed statistical analyses, and M.T. wrote the paper with input from all authors. All authors read and approved the final manuscript.
